# Infrared spectroscopy refines chronological assessment, depositional environment and pyrolysis conditions of archeological charcoals

**DOI:** 10.1038/s41598-020-69445-6

**Published:** 2020-07-24

**Authors:** E. Smidt, J. Tintner, O. Nelle, R. R. Oliveira, R. Patzlaff, E. H. Novotny, S. Klemm

**Affiliations:** 1 Institute of Physics and Materials Science, University of Natural Resources and Life Sciences, Peter Jordan Straße 82, 1190 Vienna, Austria; 2 State Office for Cultural Heritage Baden-Württemberg, Tree-ring lab, Fischersteig 9, 78343 Gaienhofen-Hemmenhofen, Germany; 3 Departamento de Geografia e Meio Ambiente, PUC-Rio Rua Marquês de S. Vicente, 225, Rio de Janeiro, RJ 22451-900 Brazil; 4 Anthropological Collections at the Archaeobotanical and Landscape Lab of the National Museum, UFRJ, Rio de Janeiro, RJ Brazil; 5 Embrapa Soil, R. Jardim Botânico, 1024, Rio de Janeiro, RJ 22460-000 Brazil; 6 Archaeology & Communication, Lammgasse 3/12, 1080 Vienna, Austria

**Keywords:** Biogeochemistry, Materials science

## Abstract

Based on infrared spectral characteristics, six archeological sample sets of charcoals from German (5) and Brazilian (1) sites, covering the time span from the nineteenth century CE to 3950 BCE, were compared to a chronological (present to the fifteenth century BCE) series of Austrian charcoals. A typical chronological trend of several bands (stretch vibrations: O–C–O of carboxylates at 1,585–1,565 and 1,385–1,375 cm^−1^, C–O carboxylic acids at 1,260–1,250 cm^−1^) that indicate oxidation and subsequently increasing hydrophilicity (O–H stretch vibration at about 3,400 cm^−1^) was also contained in the archive samples. Three sample sets fit in the typical band development according to their age. For three sample sets this conformity was not observed. Despite the age of two sample sets (3950–2820 BCE), most charcoals were assigned to the Modern Period. Apart from the high degree of carbonization, anaerobic depositional conditions over a longer period of time seem to contribute to the surprising conservation. Non-removable mineral components in charcoals, as observed in a third sample set, strongly influence infrared band intensities and positions of organic compounds. The role of inorganic components in terms of charcoal aging, and the information we can obtain from spectral characteristics in an archeological context, are discussed.

## Introduction

Black carbon (BC), a product of incomplete combustion, comprises a wide range of charred organic materials with different chemical and physical properties. Charcoal represents a main component. Apart from its function as remains in the historical and archeological context, its behavior, stability and turnover under different environmental conditions are the focus of numerous investigations in diverse research fields.

Due to its recalcitrance, BC is considered to be a relevant sink in the carbon cycle^1^. Stable and relatively persistent BC is found in deep marine sediments^2^, at archeological sites^3,4^ and as component of Chernozems^5,6^. Charcoals are archeological evidences for former land use and deforestation^7^ and support the reconstruction of the long-term development of forest ecosystems^8,9^, forest fires^10–12^ and anthropogenic activities associated with fire^13^. Chemical changes in soils due to the aging of pyrogenic carbon from slash-and-burn practices and the evolution of stable carbon stocks were observed in the Fujian Province^14^.

The aim to quantify the stable carbon pool in soils and sediments and to understand the behavior of black carbon, its residence time, degradation, turnover rates and the associated influencing factors in the environment has prompted many studies, both lab and field experiments. These have led to apparently contradictory observations due to the individual chemistry of pyrogenic organic matter and different storage conditions in soils in terms of temperature, moisture and oxygen access^15^. Some fundamental facts can be deduced from numerous experimental set-ups: feedstock composition, production temperature/carbonization degree and environmental conditions, e.g. soil properties^16,17^ are the main influencing factors for black carbon longevity or short-term degradation. These findings were compiled in review articles^18,19^, including uncertainties regarding the stocks and stability of BC, especially in boreal regions^20^. Litterbag experiments during one year in the tropical rainforest confirmed that extensive aromatization due to higher pyrolysis temperatures led to reduced degradability. Furthermore, non-lignocellulosic materials were more susceptible to degradation^21^. Specific formation conditions result in chemical and physical differences between the pyrogenic carbon fraction in biochars and wildfires^22^. Charcoals of low carbonization degree are scarcely found in the environment after only 20 years^23^. Other effects besides degradation, e.g., additional wildfires^24^, erosion and translocation into deeper soil horizons^25^ can also cause charcoal remains to disappear. Different approaches of laboratory and field incubation experiments to evaluate the stability of biochar in the environment were the basis for degradation models^26^. The quality of BC fractions and erosion should be included to improve modeling of the fate of BC in soils^27^. Degradability and quality are the main prerequisites for the function and interaction of biochar in soils^28^, such as adsorption behavior^29^, pore size and pore size distribution^30^ and the effects on plant growth^31–33^.

A variety of analytical methods and experimental approaches have been applied to investigate the chemistry of charcoals and alterations in field or lab experiments. Elemental analyses of C, O and H^34^ and their ratios in the van Krevelen diagram demonstrate the relation between rising carbon content and increasing temperatures^35,36^. The O:C ratio of fresh biochar seems to be a reliable parameter for its stability in soils^18^. The determination of benzenepolycarboxylic acids, a molecular marker for BC, is one of the established methods for quantification of BC in different environments, e.g. soils^37^ and marine sediments^38^.

Analyses of isotopes can trace the fate of BC from its origin as well as elucidate its behavior and residence time^39^. Removal of contaminants (e.g. non-pyrogenic carbon) might be relevant for the characterization of pyrogenic carbon. In this context, ^13^C NMR spectra were used to compare different pretreatments and the efficiency of decontamination^40,41^. For a mass balance of stable carbon, hydrogen pyrolysis was applied to the litterbag samples^21^ prior to the ^13^C NMR analyses in order to differentiate the stable aromatic carbon fraction from exogenous organic materials, incorporated during exposure in the environment^42^. The formation of new nitrogen heterocyclic components was observed during thermal oxidation of peat samples using ^13^C- and ^15^N-NMR spectroscopy^43^. ^13^C NMR and FTIR spectroscopy were applied to track the progressive dichromate oxidation of charcoals, produced at different temperatures, and natural charcoals, altered in the environment. They revealed that polyaromatic structures formed at temperatures > 400 °C are highly resistant to oxidation, whereas aged samples are more susceptible^17^. Chemical alterations of charcoals along the aging process over several centuries were revealed by their infrared spectral patterns^44^.

The formation of organized polyaromatic domains in charcoals is a basis for reflectance measurements that allow the reconstruction of the production temperature of modern and archeological charcoals formed at 400 °C and above^35,45^. Aging in the environment leaves characteristic mechanical properties of charcoals that can support the identification of original wood species^46^.

Based on previous investigations that have shown a characteristic trend of infrared spectral features with age under similar environmental conditions, it was the objective of this study to corroborate this development using additional sample sets of historical and archeological charcoals from archives (from the nineteenth century CE to 3950 BCE). The classification into an existing chronological series of reference charcoals that cover the time span from the present to a period from the fifteenth to the thirteenth century BCE should verify the conformance of their spectral signature with historical, dendrochronological and/ or ^14^C dating. Discrepancies between the spectral signature and the determined age are discussed in the context of literature regarding the influencing factors of aging.

## Results and discussion

The relevant bands that were used for sample evaluation are compiled in Table 1. Band positions are indicated according to Smith^47^ and Guo and Bustin^48^. The list is limited to visible band maxima. Aging/oxidation lead to interactions (e.g. H-bonds) and subsequently to broadening of bands^49^. Strong bands of inorganic components overlap smaller organic bands. Nevertheless, underlying features are included by multivariate data analysis of the spectral pattern. Data analyses were performed with selected wavenumber regions.

**Table 1:** Wavenumber position and assignment of functional groups.

Wavenumber (cm^−1^)	Molecular vibration	Functional group
3,500–2,500	O–H stretch	Carboxylic acids, water
3,000–2,800	C–H stretch	aliphatic
3,050–3,030	C–H stretch	aromatic
1,730–1,700	C=O stretch (aliphatic)	Carboxylic acids
1,710–1,680	C=O stretch (aromatic)	Carboxylic acids
1,610–1,560	C=C vibration (aromatic)	aromatic ring
1,585–1,565	O–C–O stretch (asymm.)	Carboxylates
1,385–1,375	O–C–O stretch (symm.)	Carboxylates
1,260–1,250	C–O stretch; O–H bending	Carboxylic acids
~ 1,030	Si–O-Si stretch (asymm.)	Silicates
1,060–1,030	C–O stretch (aliphatic)	Ethers and Alcohol
894, 823, 758	C–H (deformation)	

### Stepwise procedure in the interpretation of sample sets from additional sites

Principal Component Analysis (PCA) of infrared spectra (wavenumber regions of 4,000–2,400 cm^−1^ and 1,800–400 cm^−1^) is performed for all samples (reference samples and dated sample sets). If their scores correspond to the age determined by the reference method, it is indicative that alteration proceeds according to the spectral pattern of the reference samples. Otherwise, further investigations are necessary as described below.

### Trend of the spectral pattern of natural charcoal aging

**Figure 1:**
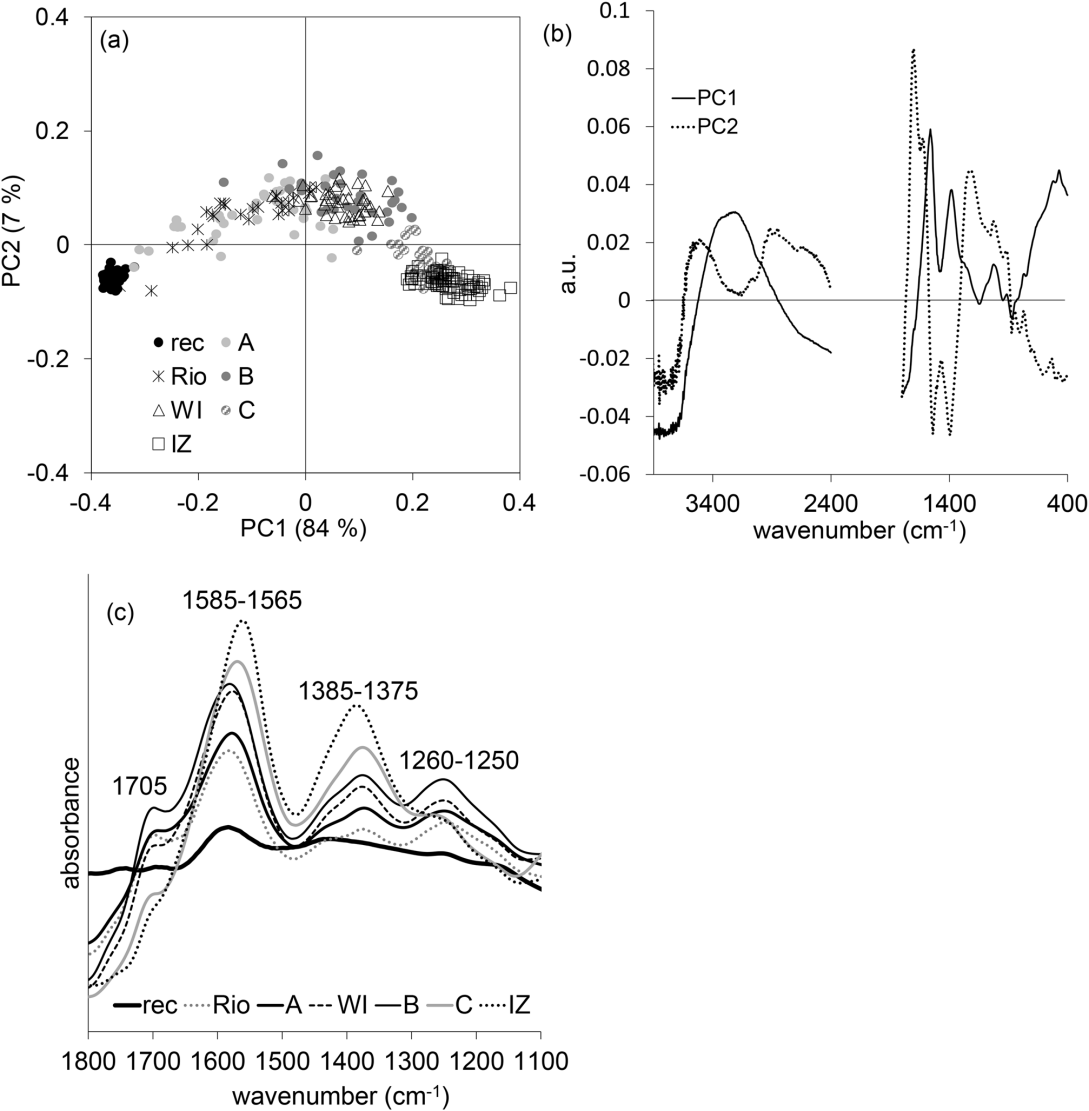
(**a**) Scores plot and (**b**) corresponding loadings plot of the PCA based on the wavenumber regions 4,000–2,400 cm^−1^ and 1,800–400 cm^−1^, (**c**) average spectra (wavenumber range 1,800–1,100 cm^−1^) of Austrian reference samples (rec, A_1800 CE_, B_13th–early 15th cent. CE_, C_15th–13th cent. BCE_) and samples from Brazil (Rio_18th–19th cent. CE_) and Germany (WI_15th–17th cent. CE_ and IZ_3280–3250 BCE_).

Due to the determined age, it can be assumed that all samples were pyrolyzed at temperatures > 400 °C. Charcoals produced at lower temperatures are more affected by microbial degradation as shown by incubation^50^, field experiments^42^ and chemical oxidation^17^. During the aging process a common succession of the spectral changes is noticeable. Figure 1a and b display the PCA (4,000–2,400 cm^−1^ and 1,800–400 cm^−1^) of reference samples from Austria (recent, A, B, C) and sample sets of charcoals from Brazil (Rio) and Germany (Wittnau WI and Iznang IZ). Despite the heterogeneity within the group, their scores in PC1 are proportional to age. The loadings plot (Fig. 1b) clearly reveals the relevance of the organic bands with regard to the aging process.

The average infrared spectra of each sample set are shown in Fig. 1c to support the loadings interpretation. They feature the characteristic development of relevant bands in the region from 1,800 cm^−1^ to 1,100 cm^−1^: the increase in intensity of the carboxylate bands (1,585–1,565 cm^−1^ and 1,385–1,375 cm^−1^) and the concomitant increase, followed by a relative decrease in the oldest samples, of the carboxylic acid bands at about 1705 cm^−1^ and 1,260–1,250 cm^−1^. This decline is observed starting from the fifteenth to the thirteenth century BCE (reference samples “C”). The samples from Brazil (Rio), Wittnau (WI) and Iznang (IZ) fit in the series according to their determined age. Emerging functional groups and changes of band intensities in the carboxylic/carboxylate region are paralleled by a corresponding increase of the O–H stretch band in the spectral region 3,500–2,500 cm^−1^, which is in accordance with the increasing hydrophilicity due to oxidation (as shown in Fig. 3). Despite different sampling sites (Germany, Brazil) and therefore different climatic conditions, the oxidation process follows a useful regularity. The degree of carbonization seems more important than some differences in the environment, as confirmed by litterbag experiments, where degradation was generally highest in 500 °C chars and lowest in 300 °C chars, independent of storage conditions such as soil surface, litter, or layer of limestone chips^42^. Changes regarding drought, humidity and temperatures might be counterbalanced over the long-lasting residence time in the environment. Nevertheless, some environmental conditions have a strong impact on alteration or preservation^15^. Heterogeneity within groups is pronounced from the nineteenth to the thirteenth century CE, whereas older groups become more uniform. Over a period of millennia, the relevance of individual degrees of carbonization or environmental exposure abates and only samples with high degrees of carbonization and appropriate environmental conditions remain.

### Archeological sites Bodnegg and Olzreute

**Figure 2:**
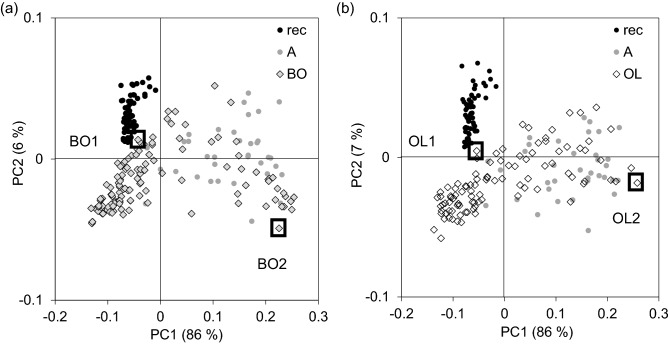
Scores plot of the PCA based on infrared spectra (wavenumber regions 4,000–2,400 cm^−1^ and 1,800–1,100 cm^−1^) of reference samples (rec, A) and (**a**) samples from Bodnegg (BO) and (**b**) samples from Olzreute (OL); squares indicate samples (BO1, BO2, OL1, OL2) for single spectra (Fig. 3) and ^14^C analyses (see below).

Two sample sets (Bodnegg and Olzreute) do not fit in the series of reference samples in terms of their spectral features. Despite the age of several thousand years (3950–3650 BCE and 2900–2820 BCE, respectively), sample positions in the scores plot of the PCA (not shown), based on all reference samples and the wavenumber regions 4,000–2,400 cm^−1^ and 1,800–400 cm^−1^, are close to recent samples (rec) or overlap samples from reference set “A” (about 1800 CE). This first PCA was the basis for the second PCA (Fig. 2), which emphasizes these two relevant periods. This second PCA used the wavenumber regions 4,000–2,400 cm^−1^ and 1,800–1,100 cm^−1^. The wavenumber region from 1,100 to 400 cm^−1^ was excluded, as mineral compounds were not visible in the spectra.

**Figure 3:**
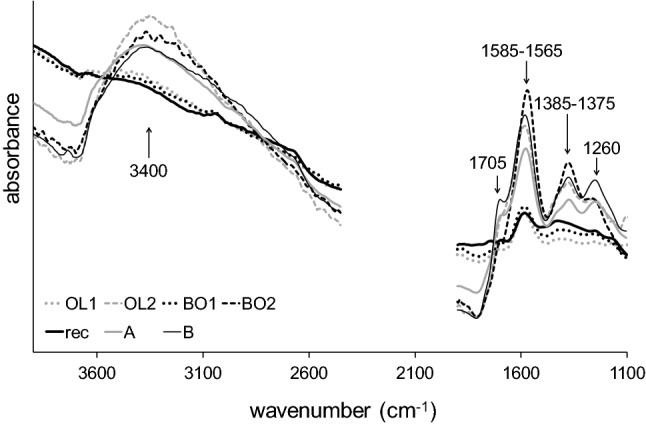
Average infrared spectra (wavenumber regions 4,000–2,400 cm^−1^ and 1,800–1,100 cm^−1^) of reference samples (rec, A, B) and single spectra of marked samples (Fig. 2) from Bodnegg (BO1 and BO2) and Olzreute (OL1 and OL2).

According to their position in the scores plot, close to either recent samples (rec) or reference samples “A”, single spectra of the marked samples in Fig. 2 from both groups (BO1 and BO2, OL1 and OL2) are displayed in Fig. 3. The wide variability of intensities in the carboxyl/carboxylate region indicates different partial oxidation degrees among different BO and OL samples (within-group variation).

As the spectra of BO2 and OL2 feature properties of reference samples C (increase of the bands at 1,585–1,565 cm^−1^ and 1,385–1,375 cm^−1^ and the concomitant decrease of the bands at 1,705 cm^−1^ and 1,260 cm^−1^), a SIMCA (Soft Independent Modeling of Class Analogy) was calculated to find out the membership of the samples.

Most of the samples are located in the area “neither–nor”, but closer to the class “rec” than “C”. Only 7 out of 125 samples from Bodnegg, and none from Olzreute, are assigned to the class “C”, as would be expected from their determined age.

The similarity to recent samples or reference samples “A” (i.e., low degree of partial oxidation) indicates some protective mechanism from aging or high charcoal recalcitrance, which could be provided by a high degree of carbonization. The minor evidence of aging and the discrepancy between the indicated age and the spectral signature required additional investigation.

**Figure 4:**
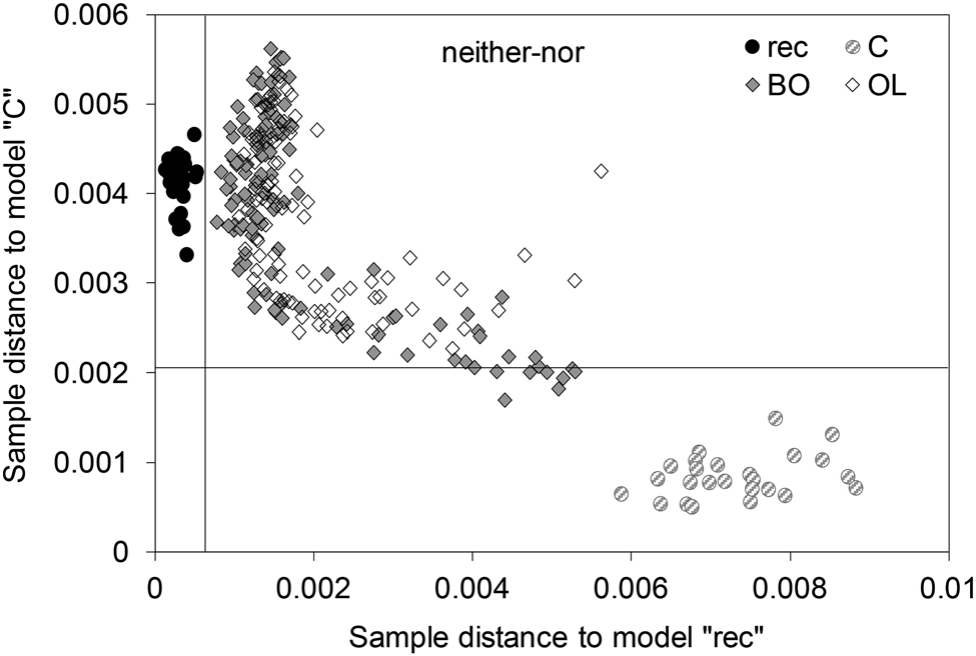
Coomans plot representing the membership of samples from Bodnegg (BO) and Olzreute (OL); classification by a SIMCA model based on infrared spectra (wavenumber regions 4,000–2,400 cm^−1^ and 1,800–1,100 cm^−1^) with defined classes “rec” and “C”_15th–13th cent. BCE_; significance level 5% (black lines).

The scores plot of the PCA (Fig. 2) and the Coomans plot (Fig. 4) reveal the heterogeneity within these groups (BO and OL), which raises the question of whether charcoals with a wider range of age coexist in the same sites. In such cases FT-IR spectroscopy provides advantageous information as it allows analyses of huge sample sets due to low costs and a rapid procedure. The spectral feature can be used for sample screening to confirm previous dating results or to initiate additional investigations. Two samples from each site, Olzreute and Bodnegg, with the highest distance in the scores plot (see squares in Fig. 2) were subjected to ^14^C-dating, which confirmed the same age for both contrasting BO- and OL-samples. Therefore, we can conclude that a high degree of carbonization and/or special environmental conditions are responsible for the preservation.

**Figure 5:**
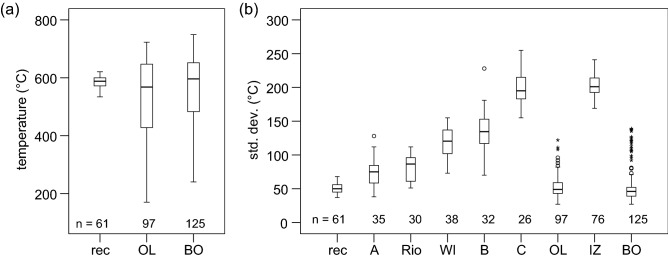
Boxplots representing (**a**) the temperature prediction of recent samples and samples from Bodnegg (BO) and Olzreute (OL) and (**b**) the standard deviations of temperature prediction for all charcoal sample sets.

In the next step, the carbonization temperature that the wood was exposed to, was determined based on spectral characteristics using an established prediction model^51^. It has to be emphasized that the prediction model has been calibrated with fresh charcoals from laboratory experiments^51^ and applied on recent traditional kiln processes^52^. The aging effect is not considered in the model. Prediction results for the current sample sets are presented in Fig. 5a and indicate that many charcoal samples from Olzreute (OL) and Bodnegg (BO) were subjected to similar temperatures as kiln samples^52^. Comparison of standard deviations of all sample sets (Fig. 5b) confirms that the application of the temperature prediction model is limited to charcoal samples that are similar to recent charcoals. High standard deviations indicate that the material departed from material characteristics of the calibrated recent charcoals.

It has to be emphasized that the sample set “rec” comprises samples with a high carbonization degree. It is not evident that such carbonization conditions can be presupposed for all pyrolysis processes to which charcoals were subjected at archeological sites. Reflectance measurements might provide more information about the production temperature, at least for > 400 °C^35,48^. The boxplots show that most samples BO and OL had been exposed to temperatures > 400 °C, half of them even > 580 °C corresponding to high thermal alteration.

Apart from the high degree of carbonization in the BO and OL samples, environmental conditions have to be considered. According to the archeological information about the sampling sites, charcoals from Bodnegg and Olzreute were embedded in a permanently wet peat. As oxygen availability and accessibility are essential factors for degradation, anoxic environments such as waterlogged ecosystems, peats and river sediments foster preservation^15^. Anaerobic environmental conditions over longer periods of time, together with the high degree of carbonization, seem to be responsible for the good state of preservation. Lab experiments with a strong chemical oxidative reagent revealed a considerable resistance of charcoals produced at 600 °C against oxidation^17^.

### Sample set with partially high content of mineral compounds (site Speckhau)

**Figure 6:**
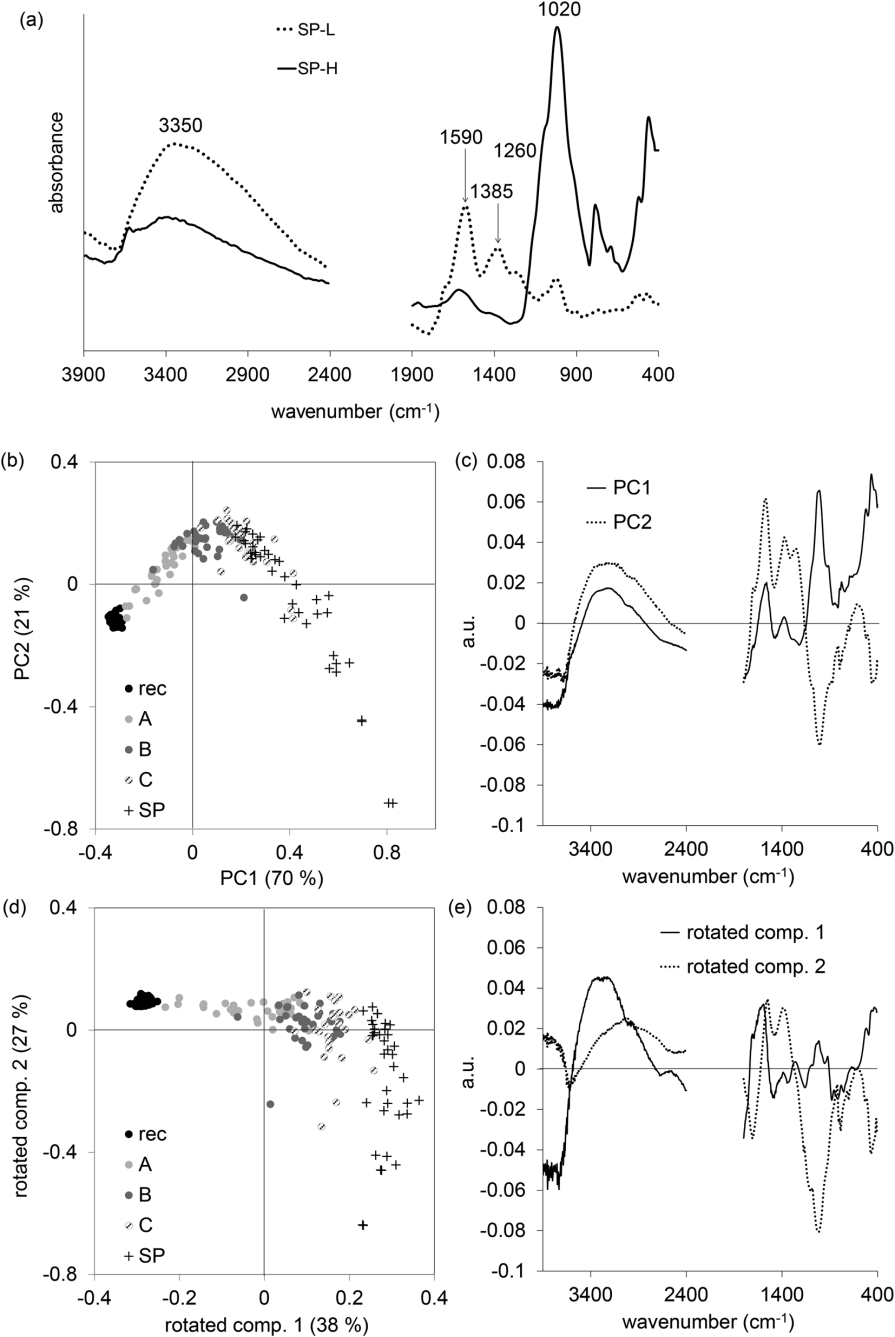
(**a**) Average FTIR-ATR spectra of samples with low (SP-L) and high (SP-H) mineral content; (**b**) scores plot of the PCA based on infrared spectra (wavenumber range 4,000–2,400 cm^−1^ and 1,800–400 cm^−1^) for all samples (SP) and reference samples (rec, A, B, C); (**c**) corresponding loadings plot of the 1^st^ and the 2^nd^ PC; (**d**) scores plot of the Varimax-Rotation based on infrared spectra (wavenumber range 4,000–2,400 cm^−1^ and 1,800–400 cm^−1^) for all samples (SP) and reference samples (rec, A, B, C); (**e**) corresponding loadings plot of the rotated component 1 and rotated component 2.

The spectral pattern of the sample set from Speckhau (SP) conspicuously indicates the environment where the charcoals were buried. These charcoals originated from a tumulus and were embedded in a mineral matrix. Mineral components (silicates, clay) had permeated the pores and could not be removed without additional chemical methods. According to Huisman et al.^53^, who investigated remains of a Neolithic settlement, alkalinity is a main factor for charcoal alteration and pedological processes that lead to clay coatings. Figure 6a displays the average spectra of both samples containing high mineral contents (SP-H) and samples with a low content (SP-L).

The high mineral content with intense infrared bands (e.g. Si–O at about 1,030 cm^−1^)^54^ obliterates other bands, with organic indicator bands disappearing almost completely. In 21 (SP-L) out of 40 samples the characteristic indicator bands of aging are at least visible. The PCA (Fig. 6b) based on infrared spectra of the whole sample set in the wavenumber range 4,000–2,400 cm^−1^ and 1,800–400 cm^−1^ illustrates the conspicuous heterogeneity due to different portions of mineral components. The corresponding loadings plot (Fig. 6c) of the 1^st^ and the 2^nd^ Principal Component (PC) reveals the spectral regions that are responsible for the main variance (explained variance by PC1 and PC2: 74% and 18%, respectively). Besides the characteristic spectral regions that represent the aging process (> 1,200 cm^−1^), the dominant contribution of mineral components with atom pairs with large reduced mass, such as Si–O, Al–O, Fe–O etc., resulting in stretching bands at low wavenumbers (region < 1,200 cm^−1^) is obvious. Charcoals with low contents (SP-L) overlap reference samples “C” and pretend an earlier period.

Although the classification into the chronological series is not possible due to the strong influence of mineral components, we can obtain some relevant information about these charcoals by means of spectral data and multivariate analysis: knowledge of the mineral environment, the heterogeneity within the group and the characteristic development of indicator bands that reveal the oxidation process.

Figure 6 displays the PCA with the same settings as in Fig. 1. Rotating the PCA (Fig. 6d, e), it is possible to isolate both sources of variation: the aging in the component 1 and the mineral contaminants in the component 2. However, the estimated age for SP samples, based on their scores in component 1, is overestimated (appearing as “C” or even older).

Ex post we can only hypothesize which factors were responsible for the accelerated aging process. Alkaline environments promote the fragmentation of charcoals and facilitate further alteration^13,45,53^. For the charcoals under limestone treatment, a lower effect of alkaline conditions was observed as expected^42^.

The question of why a part of the sample set SP incorporated more mineral components can only be answered by some assumptions. The anatomical structure of wood species might be a reason for the different ingress of mineral components^46^, charcoal burying and incorporation in the soil, and clay migration during pedogenesis. However, a close exposure to mineral components, especially to clayey materials, influences the aging process. This fact has also been observed for wood^55^. Rechberger et al.^56^ documented an accelerated aging process for biochar in acidic soils. Furthermore, a lower degree of carbonization might have led to the accelerated aging^15,44^ The simple previous inspection of the infrared spectra and identification of a large amount of minerals preclude the use of these spectra in the current models. New models for this kind of samples could overcome this limitation.

## Conclusions

Infrared spectroscopic investigations of charcoals combined with multivariate data analysis have shown that the method provides useful information on their natural aging process, which takes place in a typical way under similar environmental conditions. The characteristic pattern can be related to their age determined by other methods (^14^C dating and dendrochronology). The question is whether this fact can be used in the future as an additional dating tool. Deviations of this general pattern are observed if the degree of carbonization, environmental conditions and the ambient matrix disturb the steady transformation of the material. The discrepancy between the age determined by dating methods and the spectral features indicates additional influencing factors on the aging processes. Therefore, sampling of archeological charcoals should always be paralleled by a comprehensive survey of the sampling site, including a detailed environmental, especially pedological, report to support data interpretation. The infrared spectra alone already provide clues to some of these outliers, such as high carbonization degree and embedded mineral contaminants. In the future, if age and carbonization degree are well known, FTIR spectroscopy can be expected to provide profound information on the long-term soil conditions.

Due to the advantages of FTIR spectroscopy mentioned above, it is possible to characterize huge sample sets. Based on multivariate evaluation, the homogeneity of charcoal samples from a sampling site can be checked. In this case, infrared spectroscopy is a useful tool for a preliminary assessment in order to select additional, more expensive analyses. Future work will have to separate spectral characteristics, describing the degree of carbonization^51^ and those indicating the aging processes^44^.

In further steps, prediction models for the age of charcoal based on spectral features might be worked out, if carbonization degree and field conditions are known.

## Materials and methods

### Sample sets

Details of charcoal samples, comprising reference samples from Austria^44^ and samples from Germany and Brazil, are compiled in Tables 2 and 3. Charcoals were not excavated or dated specifically for this study.

**Table 2:** Sample ID, origin and age of reference samples^44^ and new sample sets.

Sample ID	Origin	Age^a^	No. of samples
rec	Rohr im Gebirge, Austria	recent	61
A	Eisenerz, Austria	about 1800 CE	35
B	Eisenerz, Austria	13th–early 15th cent. CE	32
C	Eisenerz, Austria	15th–13th cent. BCE	26
**Additional sample sets**			
Rio	Rio de Janeiro, Brazil	18th–19th cent. CE	30
WI	Wittnau, Germany	15th–20th cent. CE	38
IZ	Iznang, Germany	3270–3250 BCE	76
BO	Bodnegg, Germany	3950–650 BCE	125
OL	Olzreute, Germany	2900–2820 BCE	97
SP	Speckhau, Germany	400–300 BCE	40

^a^Dated by means of ^14^C and/or dendrochronology.

**Table 3:** Origin of samples and site conditions.

Point of origin	ID	History of charcoal origins of and site description
**Austrian reference materials (Smidt et al.**^44^**)**		
Rohr im Gebirge	recent	Rectangular charcoal hearths
Eisenerz	A	Rectangular charcoal hearths
	B	A charcoal production pit
	C	Copper smelting site
**Brazilian sample set**	RIO	historic charcoal kilns in the suburb of Rio de Janeiro City (22° 58′ 20″ S/43° 14′ 55″ W), Tijuca forest, samples originate from the first 15 cm of the soil profile Environmental conditions: Atlantic rainforest, altitude of 330 m a.s.l., mean annual precipitation around 1,070 mm (Details: Solórzano et al.^57^)
**German sample sets**		
Wittnau	WI	historic charcoal production site in the southern Black Forest^58^ with two usage periods (fifteenth to seventeenth century CE and seventeenth to twentieth century CE); soil: acidic (parent material is Gneis-Granitic) and well drained on a slope at 645 m a.s.l
Iznang	IZ	Domestic energy wood remains from fire places and remains of burnt-down houses from a cultural layer in and above permanently waterlogged conditions due to groundwater (Horgen settlement at the Untersee near Radolfzell/ Lake Constance^59^), dendrochronologically dated settlement with its beginnings at 3,270 BCE and with a second building phase around 3,250 BCE
Bodnegg	BO	Charcoals from fire places (settlement according to ^14^C-dates at the shore of a small lake during the neolithic period 3950–3650 BCE), wetland site with permanent wet conditions; samples embedded in slightly mineralized, but permanently wet peat
Olzreute	OL	Charcoals from excavated domestic fire places (Goldberg-III-settlement near Bad Schussenried in Upper Swabia dendrochronologically dated to 900 BCE^60^; one or two more building phases within the period around 2850–2820 BCE; the neolithic village was built at the shore of a small lake); samples incorporated and overgrown by peat layers after silting up of the lake
Speckhau	SP	Tumulus mound in a mineral, well-drained soil environment, near the Celtic settlement Heuneburg in Upper Swabia (constructed 400–300 BCE)

### Sample origin and site conditions

Table 3.

### Sample preparation

The size of collected charcoal particles was 5 to 15 mm. Charcoal particles from archives were dried at 65 °C. Attached mineral components were manually removed from the surface of charcoal particles using a tweezer and scalpel with the help of a magnifying glass. The samples were stored in small sealed glass jars. For infrared spectroscopic analyses, charcoals were milled with pestle and agate mortar to a particle size < 200 µm and stored in Eppendorf vials at room temperature.

### Fourier transform infrared (FT-IR) Spectroscopy and data evaluation

Spectra were recorded in the mid wavenumber range from 4,000 to 400 cm^−1^ using the attenuated total reflection (ATR) mode. Instrument: Tensor 27 (diamond crystal of the BRUKER Helios FTIR micro sampler with a spatial resolution of 250 µm). Three to four replicates per sample were recorded (32 scans at a spectral resolution of 4 cm^−1^), corrected against ambient air as background and unit vector normalized with the integrated software (OPUS 7.2). The averages of normalized replicates were subjected to data evaluation using the Unscrambler X 10.1 (CAMO). The wavenumber regions 4,000–2,400 cm^−1^ and 1,800–400 cm^−1^ or 4,000–2,400 cm^−1^ and 1,800–1,100 cm^−1^ were selected for Principal Component Analyses (PCA) and SIMCA (Soft Independent Modeling of Class Analogy). SIMCA provides the classification of new samples to previously defined classes regarding spectral characteristics (or other parameters). The membership of assigned samples is based on conformity or similarity to the determined classes with a given level of significance^61^. The prediction of pyrolysis carbonization used a model published in Tintner et al.^51^ Rotation of PCA was performed using the Varimax criterion^62^.

## Data Availability

Original data are available upon request.
